# Athletes with channelopathy may be eligible to play

**DOI:** 10.1007/s12471-018-1077-5

**Published:** 2018-02-06

**Authors:** N. M. Panhuyzen-Goedkoop, A. A. M. Wilde

**Affiliations:** 1 Heart Centre, Department of Clinical and Experimental Cardiology, Academic Medical Centre, Amsterdam, Amsterdam, The Netherlands; 2 Sports Medical Centre Papendal, Arnhem, The Netherlands; 3 Radboudumc Nijmegen, Nijmegen, The Netherlands

**Keywords:** Athlete, Channelopathy, Eligibility, Sudden cardiac death, QTc interval, Preparticipation screening

## Abstract

The European and Bethesda recommendations roughly state that any athlete with channelopathy is not eligible to participate in sports on a presumed risk of potentially life-threatening ventricular tachycardia or fibrillation. However, eligibility decision-making on a presumed risk of ventricular tachycardia or fibrillation is debatable. Channelopathies are primary electrical cardiac disorders and are usually transmitted as an autosomal dominant trait. Some of the channelopathies are potentially fatal in relation to exercise and predispose to life-threatening cardiac arrhythmias including ventricular tachycardia or fibrillation. Exercise, swimming, body heating and electrolyte depletion can all act as a trigger of ventricular tachycardia or fibrillation in channelopathy. However, new research mentioned a very low incidence of ventricular tachycardia or fibrillation in athletes with channelopathy challenging the decision of disqualification. Recently, the American recommendations for sports participation in athletes with a cardiovascular disorder have updated their eligibility decision-making.

In this manuscript we describe the signature features of the electrocardiogram changes in channelopathies and we argue that new research data should allow for the introduction of more liberal eligibility decision-making for sports participation in athletes with channelopathy, not only in the United States but also in European countries.

## Introduction

Channelopathies are inherited primary electrical disorders without structural abnormalities. Channelopathies are rare and usually transmitted as an autosomal dominant trait. The four major channelopathies are long QT syndrome (LQTS), short QT syndrome (SQTS), catecholaminergic polymorphic ventricular tachycardia (CPVT) and Brugada syndrome (BrS) [1, 2]. Channelopathies are identified by a 12-lead electrocardiogram (ECG) or genotyping [1–4]. Some of the channelopathies are potentially fatal in relation to exercise and predispose to life-threatening cardiac arrhythmias including ventricular tachycardia or fibrillation (VT/VF). The prevalence of sudden cardiac death (SCD) events in American young competitive athletes due to channelopathy is 4%, including 3.6% LQTS and 0.4% BrS [3, 5]. There are certain specific triggers provoking VT/VF, such as exercise (LQTS, SQTS, CPVT), increased vagal tone (SQTS, BrS), immersion in cold water (LQTS), hyperthermia/fever (LQTS, BrS), and electrolyte disturbances [4–7]. In this issue of the Netherlands Heart Journal, Zorzi et al. describe the eligibility in cardiomyopathies—hypertrophic cardiomyopathy and arrhythmogenic cardiomyopathy—that are inherited cardiac disorders with structural abnormalities predisposing to fatal cardiac arrhythmia during exercise [8].

The European and Bethesda recommendations state that roughly every athlete with channelopathy is not eligible to participate in sports on a presumed risk of VT/VF [9–12]. However, eligibility decision-making on a presumed risk of VT/VF is debatable. Athletes with, for instance, genotype-positive channelopathy do not always develop the phenotypic expression of the disease—i. e. symptoms, ECG findings of channelopathy—leading to VT/VF and can probably not be disqualified on the gene positive finding alone. Besides, new research mentioned a very low incidence of VT/VF in athletes with channelopathy challenging the decision of disqualification [13–16]. Furthermore, the American recommendations published in 2015 are more liberal in their eligibility decision-making in inherited cardiovascular disorders (CVD) [17–19].

In this manuscript we describe the signature features of the ECG changes in channelopathies and we argue that new research data should allow for the introduction of more liberal eligibility decision-making for sports participation in athletes with channelopathy, not only in the United States but also in European countries.

### Definitions

An athlete is an individual exercising regularly in competition or leisure-time aiming at improving him/herself. A young athlete is he/she aged 35 years and younger.

Regular sports participation with a certain intensity induces cardiac adaptation. Dynamic sports induce dilatation of the cavities and static sports induce thickening of the myocardium. Every sports type is classified in low, intermediate and high dynamic and/or static sports [20]. Rowing, triathlon and cycling for instance are high dynamic high static sports, and golf, billiards and riflery are low dynamic low static sports [20].

## Electrocardiography in eligibility screening

A 12-lead resting ECG in eligibility screening in athletes is generally the clue to the presence of channelopathy [17, 21–26]. In LQTS the QTc interval is prolonged, and the signature arrhythmia of LQTS is the ‘Torsades de Pointes’ arrhythmia. This is a high-rate polymorphic ventricular arrhythmia characterised by a shifting electrical axis leading to syncope and sudden cardiac arrest/sudden cardiac death (SCA/SCD) [1, 2]. The cut-off point of QTc interval prolongation in LQTS is generally accepted to be ≥500 ms—using Bazett’s formula—in repeated ECGs without known cause [1, 2]. However, for unclear reasons, in athletes a lower cut-off point ≥470 ms in males and ≥480 ms in females is used [9, 10, 17, 24–27]. Basavaradjaiah et al. found a low prevalence of LQTS in elite athletes (*n* = 7/2000) with QTc interval ≥470 ms. They also found that signs of LQTS—absence of QT interval adaptation during exercise, presence of a gene mutation, QTc prolongation in first-degree relatives—were associated with a QTc interval ≥500 ms [28]. The authors concluded that a QTc cut-off point ≥500 ms can be used in athletes provided there are no signs and symptoms—dizziness, syncope—of LQTS [28]. Maybe in future recommendations the QTc cut-off point in athletes can be considered similar to the cut-off point in the overall population. The responsible ion current for the QT interval adaptation process—paradoxical prolongation—during exercise is the catecholamine-sensitive slow component of the delayed rectifier—i. e. I_Ks_. In LQTS-1, this current is not functioning optimally and hitherto aberrant QTc prolongation occurs during an increase in heart rate. In LQTS-2 and LQTS-3, I_Ks_ functions properly and the QTc interval shortens appropriately during an increase in heart rate. This is particularly noted in LQTS-3 where QT intervals are prolonged predominantly during bradycardia and are completely normal at faster rates. The QT intervals in LQTS-2 lag behind compared with normal physiological shortening.

In SQTS the QTc interval is too short and does not change during exercise [29]. The signature arrhythmia of SQTS is atrial fibrillation or VT/VF. The disease is highly fatal [29, 30]. In SQTS there is no defined cut-off point of a short QTc interval associated with potential VT/VF in athletes. The guidelines of the European Society of Cardiology (ESC) describe that SQTS is diagnosed in the presence of a QTc interval ≤330 ms [1]. Several studies observed a very low incidence of SCA/SCD (0.02–0.1%) in the overall population and in athletes with a SQTS using different QTc cut-off points of ≤320 ms or ≤300 ms [31–35]. In addition, the short QTc interval was associated with an increased risk of recurrent SCD if the QTc interval was short [35]. Furthermore, these studies—using different cut-off points—demonstrated that male and African/Afro-Caribbean athletes were more likely to have a shorter QTc interval than female and Caucasian athletes [31–35]. Based on these studies we can conclude that African/Afro-Caribbean and Caucasian athletes with a QTc interval ≤320 ms are highly suspicious of SQTS and require additional cardiac evaluation, and symptomatic SQTS athletes should be restricted from sports participation [31–35]. Furthermore, quinidine or sotalol, both prolonging the short QTc interval, can be used and implantable cardioverter defibrillator (ICD) implantation should be considered to prevent SCA/SCD [1, 30].

CPVT is the only major channelopathy with normal ECG findings at rest. The signature feature is an exercise/emotion-induced ventricular arrhythmia, often already seen in paediatric age groups (age 5–6 years) [36]. This arrhythmia typically starts with monomorphic ventricular ectopy at a certain heart rate that is quite consistent for a given patient [36]. The typical heart rate at which ventricular ectopy develops is patient-dependent and usually in the range of 90–120 bpm. In addition, any extrasystole in morphologically normal hearts not originating from the right ventricular outflow tract should raise suspicion in relevant circumstances. It becomes more characteristic with polymorphic doublets—one is enough—and pathognomonically with bidirectional VT—which can be short—inducing episodes of syncope [36]. Deterioration into VF can occur. It is generally stated that in every athlete with CPVT physical exercise should be prohibited without exception.

In BrS, recognising the typical Brugada pattern on the 12-lead resting ECG can be a challenge. However, BrS is a clear-cut electrophysiological diagnosis [37]. A diagnostic type 1 ECG is defined as a spontaneous high take-off and downsloping ST-segment elevation of more than 2 mm above the baseline J‑point in the right precordial leads (V1–3) followed by a negative T‑wave [1, 2, 37, 38]. If there is suspicion of BrS high lead placement of V1-3 in the second and third intercostal spaces may unmask a Brugada pattern. However, in a pilot study in Dutch athletes (<24 years, *n* = 350) we did not find a Brugada pattern with high lead placement (Panhuyzen, unpublished data). If there is suspicion of the Brugada pattern provocation studies with a sodium channel blocker—i. e. preferentially ajmaline or flecainide—may be considered. However, the specificity and sensitivity of the drug challenge test is increasingly questioned [39, 40]. In addition, the risk of SCA/SCD is very low (<0.05%/year) if the provocation study with a sodium channel blocker in BrS induces ventricular arrhythmia only [39, 40]. Besides, the mechanism of inducing a fatal arrhythmia in BrS with a drug challenge test is different from exercise-induced VT/VF. Furthermore, the Brugada pattern should be distinguished from Brugada phenocopies and early repolarisation [27, 37, 38, 41]. The latter is a normal finding in highly trained endurance athletes [27, 38, 41]. Brugada phenocopies are a group of heterogenous conditions with Brugada-like ECG findings, lack of symptoms suggestive of BrS, negative family history of BrS or SCA/SCD, negative drug challenge test, and negative genetic testing [37]. In addition, SCA/SCD in BrS is generally unrelated to exercise, but occurs most often during sleep. There seems to be no evidence whatsoever to exclude these individuals from sports participation [5, 6, 14–16, 40, 44, 47].

### Cardiac evaluation

The athlete is referred for additional cardiac evaluation if the ECG findings in eligibility screening raise suspicion of channelopathy and the athlete is temporarily restricted from sports participation [9, 17]. Cardiac evaluation includes—if indicated—exercise testing, Holter monitoring, echocardiography, MRI and/or, possibly, genetic counselling [9, 10, 17, 38]. An electrophysiologic (EP) study is not recommended in the evaluation of the risk of VT/VF in channelopathy (class III indication, level of evidence C), except for BrS (Class IIB indication, level of evidence C) [1, 2, 22].

Sports-specific stress testing until exertion and Holter monitoring during training sessions have the potential to capture relevant ventricular arrhythmias [38]. In LQTS-1, for instance, the key feature is a paradoxical prolongation of the QTc interval at higher heart rates. In SQTS the QTc interval remains short, and in CPVT the key feature is an increasing number of ventricular arrhythmias with on-going exercise as described above. In addition, frequent and complex ventricular ectopy (burden >1% or >2000/24hrs) in trained athletes seems to be related to structural cardiac abnormalities [42].

### Genetic counselling and family screening

Genetic testing in the proband and/or his/her first-degree family members is considered in the diagnostic evaluation in athletes with abnormal screening findings suspicious of channelopathy [43]. However, when an athlete suddenly dies, and autopsy confirms the absence of structural heart disease, genetic testing of the deceased should be considered to identify the channelopathy gene and confirm the diagnosis [43, 44]. Once the gene is identified family screening can be considered. Unfortunately autopsy studies are not performed routinely in all countries and the question regarding the cause of death remains often unsolved. We should note that a negative genetic test result does not exclude the presence of an inherited disease.

## More liberal eligibility decision-making

Current and new developments in the diagnosis and management of channelopathies probably control the risk of exercise-related cardiac events—i. e. VT/VF—better than before, suggesting that eligibility decision-making in channelopathy may be more liberal [9, 10, 13, 21]. In VF slow calcium channels as well as sodium channels are involved in the initiation of VF [45]. It is generally accepted that certain lifestyle features may increase the risk of VT/VF in athletes with channelopathy [1, 2, 17, 27, 36, 43]. QT-prolonging drugs (www.crediblemeds.org) in LQTS and drugs aggravating the disease (www.brugadadrugs.org) in BrS should be avoided. In addition, it is recommended to avoid hyperthermia and/or excessive sweating in channelopathy. The physiologic process of a higher core temperature during exercise is associated with vasodilatation and sweating to prevent the body from heating—body temperature >40 degrees Celsius. However, in exceptional cases and in exercise during fever the body temperature can increase to a dangerous level and fatal arrhythmias may occur. It is generally accepted that athletes with BrS and/or certain LQTS mutations should avoid exercise in hot conditions and during fever to prevent body heating [1, 2, 17, 27, 36, 43]. Prophylactic use of acetyl-p-aminophenol 500 mg seems controversial. Burtscher et al. measured the body temperature in a randomised cross-over trial in seven male young non-feverish athletes who used prophylactic acetyl-p-aminophenol 500 mg or a placebo two hours before exercising on a treadmill in a climate chamber at 30 degrees Celsius at an individual intensity of 70% VO2_max_ [46]. The increase in body temperature was slightly reduced by acetyl-p-aminophenol from 38.4 to 38.0 degrees Celsius and the physical performance remained unaffected [46]. However, there are no data on prophylactic acetyl-p-aminophenol in channelopathy to avoid body heating.

The recommendations of the American Hearts Association (AHA) and the American College of Cardiology (ACC) for athletes published in 2015 are the first to be more liberal in eligibility decision-making in athletes with inherited CVD [17–19]. Overall, we do agree with these recommendations, although the level of evidence is low. However, the international guidelines on the primary arrhythmia syndromes need to be considered when we want to describe more specific recommendations for athletes with channelopathy, taking into account the stage and type of the channelopathy, and the intensity and type of the sport practiced by the athlete [1, 2].

In a large Italian screening study, young athletes with LQTS—0.6% of all non-eligible athletes—were disqualified on a presumed risk of an exercise pro-arrhythmic trigger of VT/VF [37]. During follow-up no cardiac events in the disqualified athletes were reported [47]. After more than 30 years of screening (*n* = 42,386) the authors observed that only two of the LQTS cases had died suddenly [47]. Johnson et al. have studied athletic participation and LQTS-related events in a retrospective case record study in gene-positive young LQTS athletes—aged 6–40 years—who continued sports participation despite medical advice (*n* = 157) after being informed in detail about the risk of sports participation by reading the Bethesda conference guidelines, which was also agreed upon by the parents in case of a minor [13]. The vast majority of athletes were treated with β‑blocking agents (95%) and, if deemed necessary, implantation of ICD (*n* = 20). They were advised to avoid dehydration, body heating, electrolyte disturbances and QT-prolonging drugs and were instructed to carry their own automated external defibrillator (AED) in their equipment. During a mean follow-up of 5.5 years in 60 athletes participating in all sports no cardiac events were reported except for one boy. The boy had unexplained fainting during physical and emotional stress with a QTc interval 490 ms at age 7 years. When his QTc interval prolonged to >550 ms he suffered from SCA and an ICD was implanted. He had two appropriate VF-terminating ICD shocks during soccer and basketball while being non-compliant with his β‑blocking agents [13]. To date this is the only study in a large number of gene-positive LQTS patients demonstrating the relative benign nature of appropriately treated LQTS and it tends to show that disqualification on a presumed risk of VT/VF is not justified. However, it remains uncertain if an ICD provides safe protection for SCD in athletes with channelopathy [13, 14]. Colman et al. have compared case records of LQTS patients presenting with syncope (*n* = 41) in an unselected group of syncope patients (*n* = 113) presenting at the emergency department [48]. The authors concluded that a positive family history for sudden cardiac death (*n* = 21), palpitations prior to syncope (*n* = 12), syncopal episodes in supine position (*n* = 24), and syncopal episodes related to exercise (*n* = 10) and emotional stress (*n* = 21) are more common among patients with LQTS [48].

To date in SQTS there are no studies in athletes to determine the risk of VT/VF in relation to exercise.

Ostby et al. (Mayo Clinics) observed in a follow-up study of adolescent CPVT athletes (*n* = 21/63) who continued competition after shared decision-making that only three athletes suffered a cardiac event during exercise [39]. Prior to the diagnosis, 16 of the 21 athletes continuing competition had experienced CPVT-triggered events. They concluded that the risk of cardiac events in well-treated and well-informed athletes with CPVT may be acceptable, although CPVT if untreated poses a high risk of VT/VF [39]. Athletes are usually more aware of their physical well-being and the risk associated with their sports than the overall population. However, when an athlete is identified with channelopathy it is very important to discuss all the information the athlete needs for shared-decision making in the management of and recommendation for sports participation. This is probably the most important part in the sports cardiology practice.

In a review study of 18 articles, Masrur et al. indicated that there were only limited data in BrS patients describing exercise-related VT/VF and stated that the risk of fatal arrhythmias seems to be unclear [15]. Olde Nordkamp et al. have reviewed case records of BrS patients (*n* = 342) with aborted SCA (*n* = 23) or non-arrhythmic syncope (*n* = 118) [49]. The SCA was not triggered by high temperature, pain or emotional stress. Those with syncope experienced their first event at an older age (mean age 45 years) than the SCA group (mean age 20 years). The authors concluded that arrhythmic and non-arrhythmic syncopes often occur in BrS [49].

In an international ICD registry in athletes, Lampert et al. found that except for VF terminating ICD shocks (*n* = 37) no cardiac events were reported in athletes with an ICD who continued sports participation in organised (*n* = 328) or high-risk (*n* = 44) sports against medical advice [14]. In this study cohort 73 athletes had LQTS, 10 CPVT and 7 BrS [14]. VF terminating shocks occurred in two athletes with LQTS and in one with CPVT. Although the authors concluded that athletes with an ICD can participate in competitive sports without failure of VT/VF termination or physical injury, it is uncertain whether an ICD provides sufficient protection to prevent SCD related to sports participation [14].

Considering the few, but important, studies mentioned above, we would like to propose more liberal eligibility decision-making in athletes with channelopathy (Tab. 1):

**Table 1:** Proposal for more liberal eligibility decision-making in athletes with channelopathy

	Long QT Syndrome	Short QT Syndrome	CPVT	Brugada Syndrome
Asymptomatic phenotype +	QTc ≥ 500 ms : no sports	All sports	Low-intensity sports	All sports
	QTc > 470 ms (males) or >480 ms (females): lifestyle changes	Consider Quinidine or Sotalol	β-blocker recommended	All sports
Symptomatic phenotype +	Consider low-intensity sports	No sports	No sports	No sports
	In SCA β‑blocker and/or ICD	ICD recommended	ICD recommended	Consider ICD
Asymptomatic phenotype + oral drugs and/or ICD	No event in the past 3 months	No events in the past 3 months	No event in the past 3 months	No event in the past 3 months
	Consider competitive sports	Consider low-intensity to moderate-intensity competitive sports without peak exertion	Consider competitive sports	Consider competitive sports
Genotype + phenotype –	All sports	All sports	All sports	All sports
			Consider β‑blocker	
Genotype + phenotype − SCD in family	All sports	All sports	All sports	All sports
			Consider β‑blocker	
Lifestyle changes Note: athletes with ICD should avoid bodily collision and sports-inducing risk for other sports participants	Avoid QT-prolonging drugs (www.crediblemeds.org), dehydration and/or excessive sweating, electrolyte disturbances, hyperthermia, and exercise while suffering from a fever	Avoid dehydration and/or excessive sweating, hyperthermia, and exercise while suffering from a fever	Avoid strenuous exercise, stressful environment, dehydration and/or excessive sweating, electrolyte disturbances, hyperthermia, and exercise while suffering from a fever	Avoid drugs that may aggravate the disease (www.brugadadrugs.org), dehydration and/or excessive sweating, electrolyte disturbances, hyperthermia, and exercise while suffering from a fever
	LQTS1: no swimming, diving, immersion in cold water			

*CPVT* catecholaminergic polymorphic ventricular tachycardia, *ICD* implantable cardioverter defibrillators*, SCA* sudden cardiac arrest, *SCD* sudden cardiac death, + positive, – negative

### Eligibility decision-making in LQTS

1. In asymptomatic phenotype-positive LQTS with QT interval ≥500 ms sports participation is restricted, and β‑blockers are recommended.
2. In symptomatic phenotype-positive LQTS (syncope, dizziness) sports participation should be restricted and only low intensity sports (Mitchell’s classification of sports), can be considered. β‑blockers are recommended. ICD implantation is recommended in patients with previous SCA and in patients with syncope and/or VT while receiving β‑blockers [2].
3. When there are no ventricular arrhythmia events recorded with the ICD during at least 3‑month follow-up and the QTc is <500 ms recorded on repeated ECGs the physician may consider return-to-play in sports provided the athlete does not participate in swimming and diving (LQTS-1).
4. Genotype-positive phenotype-negative LQTS individuals—i. e. ECG diagnosis of LQTS without symptoms related to LQTS—are allowed to participate in all sports. In general, a family history of fatal cardiac events has not been associated with a higher risk for cardiac events in subsequent family members and in all survival analyses phenotype-negative patients (i. e. normal QTc) have a much lower risk compared with their phenotype-positive family members [1].
5. All patients with LQTS QT prolonging drugs (www.crediblemeds.org) should avoid dehydration and/or excessive sweating, electrolyte disturbances, hyperthermia, and exercise during fever. In patients with LQTS-1 swimming and diving should be discouraged because these activities are well-known triggers for cardiac events [2].

### Eligibility decision-making in SQTS

1. In asymptomatic phenotype-positive SQTS all sports are allowed [2]. Oral drugs (quinidine or sotalol to increase the QTc interval) may be considered.
2. In symptomatic phenotype-positive SQTS—survivors of aborted SCA, documented spontaneous sustained VT—low intensity sports (Mitchell’s classification of sports) may be considered. ICD implantation is recommended and if there are no cardiac events for at least 3 months participation in low-moderate intensity sports (Mitchell’s classification of sports) may be considered [2, 43].
3. In genotype-positive phenotype-negative SQTS individuals with or without at least one first-degree family member with SCD are allowed to participate in all sports.
4. All patients with SQTS should avoid dehydration and/or excessive sweating, hyperthermia, and exercise during fever. Hyperkalaemia, for instance, associated with dehydration and/or excessive sweating should probably be avoided because high extracellular potassium levels may further shorten the QT interval.

### Eligibility decision-making in CPVT

1. In asymptomatic phenotype-positive CPVT with exercise-induced or emotionally initiated polymorphic VT only low intensity sports (Mitchell’s classification of sports) are allowed and β‑blockers are recommended.
2. In symptomatic phenotype-positive CPVT (exercise-induced recurrent syncope, aborted SCA, polymorphic VT despite β‑blockers, verapamil or flecainide) sports participation is restricted to low intensity sports (Mitchell’s classification of sports). If there are no ventricular arrhythmic events during at least 3‑month follow-up low to moderate intensity sports (Mitchell’s classification of sports) may be considered.
3. In genotype-positive phenotype-negative CPVT β‑blockers are to be considered. Those using β‑blockers can be allowed to participate in all sports with the advice to avoid strenuous exercise and stressful circumstances.
4. All CPVT patients should avoid stressful environment and strenuous exercise, dehydration and/or excessive sweating, electrolyte disturbances, hyperthermia, and exercise during fever.

### Eligibility decision-making in Brugada syndrome

1. In asymptomatic BrS—i. e. spontaneous type 1 ECG—, there is no restriction for sports participation. Ventricular arrhythmia only on provocation with a sodium channel blocker would not change this recommendation.
2. In symptomatic BrS (palpitations, dizziness) all sports are allowed. If there is syncope or aborted SCA sports participation is restricted and ICD implantation should be considered. Resuming sports participation can be allowed if there are no appropriate shocks during at least 3‑month follow-up [1].
3. Genotype-positive—i. e. pathogenic SCN5a mutation—phenotype‐negative BrS with or without at least one family member with SCA/SCD are allowed to participate in all sports.
4. All patients with BrS drugs that may aggravate the disease (www.brugadadrugs.org) should avoid dehydration and/or excessive sweating, electrolyte disturbances, hyperthermia, and exercise during fever.

### Eligibility decision-making in overlap syndromes

For patients with overlap syndromes the advices relating to both disease entities (for example, LQTS and BrS) are pertinent. We have mentioned that separately.

## Conclusion

New research data should allow for the introduction of more liberal eligibility decision-making for sports participation in athletes with channelopathy. Eligibility decision-making in channelopathy should involve the opinion of cardiologists with expertise in these rare syndromes and, to date, cannot be based on a presumed risk of pro-arrhythmia related to exercise. Athletes—and their parents in case of a minor—are entitled to detailed information about the risk of sport participation and should be part of the shared decision-making process to continue sports participation.
